# Antimicrobial activity of Ib-M peptides against *Escherichia coli* O157: H7

**DOI:** 10.1371/journal.pone.0229019

**Published:** 2020-02-13

**Authors:** Sergio Prada-Prada, Johanna Flórez-Castillo, Ana Farfán-García, Fanny Guzmán, Indira Hernández-Peñaranda

**Affiliations:** 1 Maestría en Investigación en Enfermedades Infecciosas, Facultad de Ciencias de la Salud, Grupo de Investigación en Ciencias Básicas y Aplicadas para la sostenibilidad – CIBAS, Universidad de Santander, Bucaramanga, Colombia; 2 Departamento de Ciencias Naturales, Facultad de Ciencias Exactas, Naturales y Agropecuarias, Grupo de Investigación en Ciencias Básicas y Aplicadas para la sostenibilidad – CIBAS, Universidad de Santander, Bucaramanga, Colombia; 3 Programa de Bacteriología y Laboratorio Clínico, Facultad de Ciencias de la Salud, Grupo de Investigación en Manejo Clínico – CLINIUDES, Universidad de Santander, Bucaramanga, Colombia; 4 Laboratorio de Síntesis de Péptidos, Núcleo de Biotecnología Curauma (NBC), Pontificia Universidad Católica de Valparaíso, Valparaíso, Chile; Nitte University, INDIA

## Abstract

The development of new antimicrobial peptides has become an attractive alternative to conventional antibiotics due to the increasing rates of microbial drug resistance. Ib-M corresponds to a family of cationic synthetic peptides, 20 amino acids in length, that have shown inhibitory effect against the non-pathogenic strain *Escherichia coli* K-12. This work evaluated the antimicrobial potential of Ib-M peptides against the pathogenic *E*. *coli* O157: H7 using a reference strain and a clinical isolate. The Ib-M peptides showed antibacterial activity against both strains of *E*. *coli* O157: H7; the minimum inhibitory concentration of Ib-M peptides ranged from 1.6 to 12.5 μM and the minimum bactericidal concentration ranged from 3.7 to 22.9 μM, being Ib-M1 and Ib-M2 the peptides that presented the highest inhibitory effect. Time-kill kinetics assay showed a reduction of the bacterial population by more than 95% after 4 hours of exposure to 1xMIC of Ib-M1. Low cytotoxicity was observed in VERO cells with 50% cytotoxic concentration in the range from 197.5 to more than 400 μM. All peptides showed a random structure in hydrophilic environments, except Ib-M1, and all of them transitioned to an α-helical structure when the hydrophobicity of the medium was increased. In conclusion, these findings support the *in vitro* antimicrobial effect of Ib-M peptides against the pathogenic bacteria *E*. *coli* O157: H7 and prove to be promising molecules for the development of new therapeutic alternatives.

## Introduction

Pathogenic bacteria with antimicrobial resistance has become a global public health threat leading to the research and development of new antibiotics [1]. Antimicrobial peptides (AMPs) are naturally occurring small molecules, 15–20 amino acids in length, with activity towards a broad spectrum of bacteria and fungi, including multi-drug resistant bacteria and recalcitrant pathogens associated with biofilms [2]. AMPs overcome the resistance induction mechanisms of microorganisms, and hence, they are a promising alternative to current therapeutic strategies. The primary mechanism of action of AMPs involves membrane disruption, a process initiated with the attracting electrostatic interactions established between the cationic residues of the AMPs and the anionic components of the microbial cell membranes; they act by disrupting the cytoplasmic membrane or affecting intracellular targets [3,4]. AMPs are secreted by the innate immune system of animals and plants against pathogenic microorganisms. They can be isolated from different organisms such as plants, amphibians and insects, among others, while analog versions of AMPs, with equal or superior antimicrobial activity to native peptides, can be synthetically developed by solid-phase peptide synthesis [5,6]. Ib-M family is made up by analog peptides to Ib-AMP4, a group of plant-derived AMPs, which were obtained synthetically by modifying the net charge and hydrophobicity of the native peptide by inserting arginine and tryptophan residues; as a result, the Ib-M peptides had an increased inhibitory effect with respect to Ib-AMP4 against the non-pathogenic *Escherichia coli* K-12 [7]. To extend the previous findings on the antimicrobial activity of the Ib-M peptides, this work evaluated its antibacterial properties against *E*. *coli* O157: H7, the most prevalent serotype of shigatoxigenic *E*. *coli* (STEC), a well-established bacterial foodborne pathogen, whose overall incidence is estimated in approximately 2.8 million cases of acute gastrointestinal disease annually [8]. The STEC-induced illness is characterized by bloody diarrhea, hemorrhagic colitis, and complications associated with the development of hemolytic uremic syndrome [9,10].

The antibacterial activity of Ib-M peptides against *E*. *coli* O157: H7 was evaluated by determining its minimum inhibitory concentration (MIC), and minimum bactericidal concentration (MBC). Bacterial cell growth kinetics and survival kinetics were assessed within a 24 hour period, and the analysis of the secondary structure of the peptides was performed by circular dichroism. Herein, this study supports the antimicrobial activity of Ib-M peptides against pathogenic *E*. *coli*.

## Materials and methods

### Compounds

The Ib-M peptides (Ib-M1, Ib-M2, Ib-M4, Ib-M5, and Ib-M6) were used in this study. They have a cationic charge of +6 and 20 amino acids in their structure. General characteristics of peptides have already been described by Flórez-Castillo [7]. The sequences of each peptide are shown in Table 1. Ib-M peptides were manufactured by Biomatik^®^ and stock solutions were prepared in Tris-HCl buffer (10 mM pH 7.4) and stored at -80°C until used.

**Table 1:** Amino acid sequences of the peptides used in this study.

Peptide	Sequence	Ref
Ib-M1	EWGRRMMG**R**GPGRRMMR**W**W**R**-NH2	[7]
Ib-M2	EWGRRMMG**WR**PGRRMMR**W**W**R**-NH2	
Ib-M4	EWGRRMMG**RG**PGRRMMR**R**W**W**-NH2	
Ib-M5	EWGRRMMG**WR**PGRRMMR**R**W**W**-NH2	
Ib-M6	EWGRRMMG**WGR**GRRMMR**R**W**W**-NH2	

Streptomycin (STP) and gentamicin (GNT) from SIGMA-ALDRICH were used as reference antibiotics. Stock solutions were prepared in Müller Hinton Broth (MHB) before each experiment.

### Bacterial strains

Two strains of *E*. *coli* were used to determine antibacterial activity: i) The reference strain *E*. *coli* O157: H7 (ATCC^®^ 43888^™^) and ii) A clinical isolate of *E*. *coli* O157: H7 (AC188) wich was kindly donated by Dr Ana Elvira Farfán of the Universidad de Santander-UDES. The isolate was collected and characterized as described by Farfán (2017) [11]. The strains were kept cryopreserved at -80°C in Luria-Bertani Broth (LBB) with 15% glycerol. For the reactivation of the microorganisms, 50 μL of the cryopreserved material was added to 5 mL of LBB and then incubated at 35 ± 2°C from 18 to 24 hours before each test.

### Cells

Dr Liliana Torcoroma García kindly donated VERO cells (ATCC^®^ CCL-81^™^) from the Universidad de Santander-UDES. Cells were grown in RPMI 1640 medium supplemented with 10% of inactivated fetal bovine serum (iFBS) and incubated in a 5% CO_2_ atmosphere at 35 ± 2°C.

### Circular dichroism

Circular dichroism spectra were determined to examine the secondary structure of peptides. The experiments were performed using a J-815 spectropolarimeter. Spectra of peptides were measured at a concentration of 106 μM in 30 mM sodium dodecyl sulfate (SDS), 30% v/v 2,2,2-trifluoroethanol (TFE) and Tris-HCl buffer (10mM pH 7.4) using a 10mm length quartz cell at 20°C from 190 nm to 250 nm with data pitch 0.5 nm and scan speed 100nm/min.

### Antimicrobial activity

#### Minimum inhibitory concentrations (MIC)

MIC was determined using the microdilution method as described in protocol M07-A9 of the Clinical and Laboratory Standards Institute [12]. Briefly, 1:2 serial dilutions of Ib-M peptides in MHB were placed in a 96-well round-bottom plate at concentrations ranging from 100 to 0.05 μM; or reference antibiotics STP and GNT between 200 to 0.1 μM. The bacterial inoculum was prepared from a subculture of *E*. *coli* O157: H7 (ATCC 43888 or AC188) in LBB incubated for 18–24 hours at 35 ± 2°C before to the test. The bacteria suspension was diluted to 1x10^8^ colony forming units (CFU)/mL, to obtain a turbidity equivalent to 0.5 on the McFarland scale, confirmed by spectrophotometry upon reaching an absorbance between 0.08–0.1 at a wavelength of 625 nm; then a 1:200 dilution in MHB was performed to obtain a final concentration of 5x10^5^ CFU/mL. The diluted bacterial suspension was added to the 96-well plate containing the serially diluted peptides. The final volume of 200 μL per well consisted of 100 μl of the compound and 100 μL of diluted bacteria suspension. Negative and positive growth controls were performed by adding only MHB or *E*. *coli* O157: H7 with MHB to the wells, respectively. At the end of the incubation time, MIC was determined as the lowest compound concentration at which no bacterial growth was observed.

#### Minimum bactericidal concentration (MBC)

MBC was determined as described in the M26-A protocol of the CLSI, 1999 [13]. For this case, sub-cultures were inoculated onto blood agar plates by adding 100 μL from the wells treated with peptides in the MIC assay that did not show visible growth of the microorganism. Blood agar cultures were incubated at 35 ± 2°C for 18–24 hours, and the number of CFU was estimated. MBC was interpreted as the concentration of compound in which the colony count was equal to or less than 10.

#### Growth kinetics

The kinetics of cell growth of *E*. *coli* O157: H7 in the presence of the peptides were followed during 24 hours. The tests were performed with the reference strain ATCC 43888, and the antibacterial activity of the compounds was evaluated in the latency phase (lag phase) and the logarithmic phase (log phase).

For the lag phase, *E*. *coli* O157: H7 at 5x10^5^ CFU/mL with MHB in 96-well plate was treated with Ib-M peptides at a concentrations of 0.5xMIC, 1xMIC, 2xMIC in a final volume of 200 μL/well for 24 hours at 37°C. The turbidity was determined spectrophotometrically at 595 nm for samples taken at 0, 2, 4, 6, 8, 10, 12 and 24 hours.

In the log phase, the peptides were evaluated at concentrations of 0.5xMIC, 1xMIC, 2xMIC, 4xMIC and 8xMIC; the compounds were only added to the wells when the absorbance was in the 0.2–0.3 range equivalent to the 10^8^–10^9^ CFU/mL range [14]. Reference antibiotics, positive and negative growth controls were also evaluated in both growth phases.

#### Time-kill kinetics

*E*. *coli* O157: H7 (ATCC 43888) at 5x10^5^ CFU/mL with MHB in 96-well plates was mixed with 1xMIC and 2xMIC of the Ib-M1 peptide and incubated at 37°C for 0, 0.5, 1, 2, 4, 6, 8, 10, 12 and 24 hours [13]; then, serial dilutions in saline solution were made from the treated wells, and 10 μL of each dilution was dispensed as a drop in blood agar and incubated for 20 hours. After incubation time, colony counts were performed by selecting the dilution that gives 3 to 30 colonies per drop dispensed [15]. The bactericidal effect was determined by 99.9% reduction of *E*. *coli* (decrease >3 Log10 of CFU/mL) comparing to the initial inoculum concentration [13].

### Cytotoxicity

VERO cells at a concentration of 3x10^4^ cells/mL were plated in 96 flat-bottom wells and incubated for 24 hours at 37°C in a 5% CO_2_ atmosphere. Subsequently, the cells were exposed to 1:2 serial dilutions of Ib-M peptides in the range from 400 to 1.6 μM and the reference antibiotics between 3200 and 1.6 μM. Control cells were maintained without peptides or reference antibiotics. After 24 hours of incubation, VERO cells viability was determined using the MTT (3- (4,5-dimethiazol-2-yl) -2,5-diphenyltetrazole bromide) colorimetric technique (Mosmann, 1983) [16]. To do so, 20 μL/well of MTT was added at a concentration of 5 mg/mL and incubated for 4 hours. After that time, the culture medium was removed from the wells, and 100 μL of dimethylsulfoxide was added to solubilize the formazan crystals. Absorbance was determined by spectrophotometry using a wavelength of 595 nm.

### Statistical analysis

One-way ANOVA was used to compare the values obtained in the MICs or MBCs of the Ib-M peptides against *E*. *coli* O157: H7; the posthoc analysis was performed with the Sidak test using GraphPad Prism 7 software. Values of *p*<0.05 were considered statistically significant.

The 50% cytotoxic concentrations (CC_50_) were calculated by sigmoid regression analysis from the inhibition percentages using the XLFit program (^©^2019 IDBS). The Selectivity Index (SI) was calculated as the ratio between CC_50_ in VERO cells and the MICs in *E*. *coli* O157: H7 [17]. For those compounds whose CC_50_ could not be determined, the maximum evaluated concentration was used to calculate the respective SI.

## Results

### Circular dichroism

**Fig 1:**
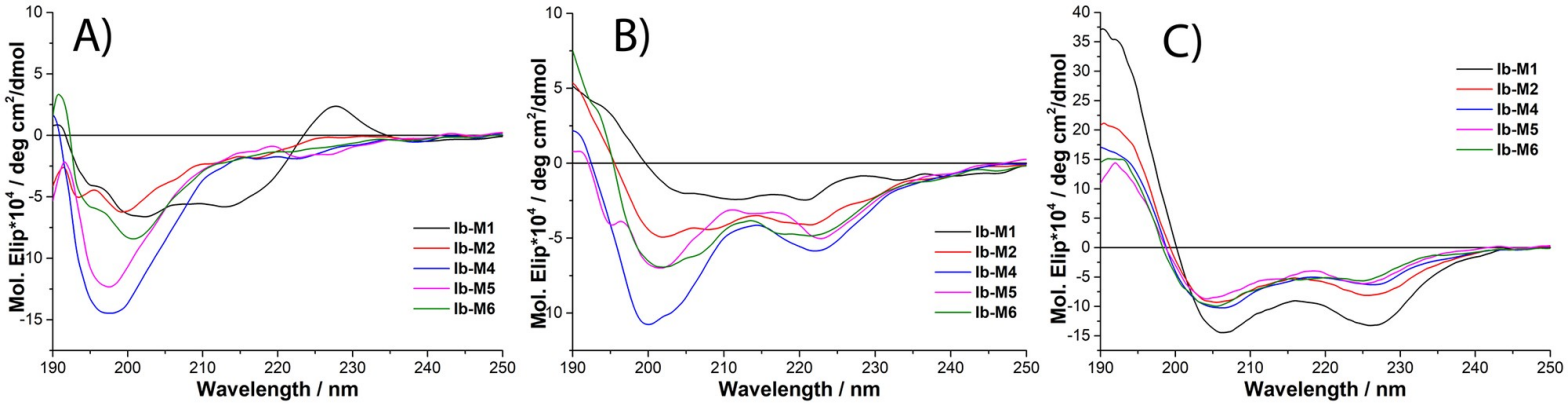
Circular dichroism spectrum of Ib-M peptides in Tris-HCl buffer (A), TFE (B) and SDS (C).

In the presence of Tris-HCl buffer, all the peptides had a random coil structure except for Ib-M1, which has a polyproline II structure (Fig 1). All Ib-M peptides showed an α-helical structure in SDS; these results suggest the peptides acquired their secondary structure in the presence of the cell membrane of *E*. *coli* (Fig 1).

### Antimicrobial activity

#### Minimum inhibitory concentration (MIC)

The MICs of the Ib-M peptides against *E*. *coli* O157: H7 were obtained in a range from 4.7 to 12.5 μM and from 1.6 to 6.3 μM for strains ATCC 43888 and AC188 respectively. In both strains, Ib-M1/Ib-M2 and Ib-M4 were the peptides that presented the highest and lowest inhibitory activity, respectively (Table 2).

**Table 2:** Minimum inhibitory concentration (MIC) and minimum bactericidal concentration (MBC) of Ib-M peptides against *E*. *coli* O157: H7.

Compound	*E*. *coli* O157: H7			
	ATCC 43888		AC 188	
	μM ± S.D.		μM ± S.D.	
	MIC	MBC	MIC	MBC
**Ib-M1**	4.7 ± 1.7	6.3 ± 0.0	1.6 ± 0.0	3.7 ± 1.3
**Ib-M2**	4.7 ± 1.7	6.8 ± 3.1	3.7 ± 1.28	4.2 ± 1.6
**Ib-M4**	12.5 ± 0.0	22.9 ± 5.1	6.3 ± 0.0	15.6 ± 10.3
**Ib-M5**	8.3 ± 3.2	15.6 ± 7.7	4.7 ± 1.7	7.3 ± 2.6
**Ib-M6**	9.4 ± 3.4	11.5 ± 7.3	4.7 ± 1.7	10.4 ± 3.2
**STP**	9.4 ± 3.4	11.5 ± 2.6	66.7± 25.8	108.3 ± 49.2
**GNT**	1.8 ± 0.6	4.7 ± 1.7	2.3 ± 0.9	4.2 ± 1.6

Each concentration was evaluated in triplicate in two independent experiments. The results are expressed in terms of the arithmetic average of each group ± standard deviation (S.D.), **STP:** Streptomycin, **GNT:** Gentamicin

In the case of strain ATCC 43888, Ib-M1/Ib-M2 showed a higher inhibitory effect than Ib-M4 and Ib-M6. The Ib-M5 activity was similar than that of the other Ib-M peptides. The reference antibiotic GNT showed higher activity than Ib-M4, Ib-M5 and Ib-M6; whereas STP had a similar activity than those peptides. Ib-M1 and Ib-M2 showed an activity similar to GNT and were more effective than STP (Table 2).

With strain AC188, Ib-M1 exhibited higher inhibitory activity than the other peptides (*p* <0.05) except with Ib-M2 (p = 0.089). To inhibit the growth of the clinical isolate AC188, STP required seven times the concentration it used with the reference strain ATCC 43888 (66.7 vs. 9.4 μM, respectively). The inhibitory behavior of GNT was similar to that observed against strain ATCC 43888 (Table 2).

#### Minimum bactericidal concentration (MBC)

The MBC values of the peptides were calculated in the ranges from 6.3 to 22.9 μM and from 3.7 to 15.6 μM for strains ATCC 43888 and AC188 respectively. The MBC values obtained by Ib-M peptides were similar; only Ib-M4 had a lower bactericidal effect when compared with Ib-M 1, Ib-M2, and Ib-M6. (Table 2). GNT had a bactericidal activity comparable to the peptides on both strains, but a superior killing activity than Ib-M4 and Ib-M5 on strain ATCC 43888. Similar to the results of the MIC test, STP required a concentration nine times higher than that used against strain ATCC 43888 to exert its bactericidal effect on strain AC188 (11.5 vs. 108.3 μM respectively) (Table 2).

#### Growth kinetics

**Fig 2:**
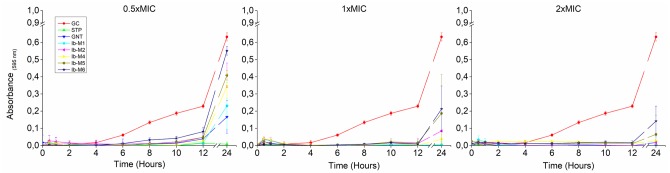
Growth kinetics of *E*. *coli* O157: H7 with Ib-M peptides in lag phase. Each concentration was evaluated in quadruplicate. Results were expressed in terms of the arithmetic average ± standard deviation. Data representative of two independent experiments with similar results is shown. **GC:** Growth Control, **STP:** Streptomycin, **GNT:** Gentamicin.

During the lag phase, growth inhibition was observed in the first 12 hours of exposure of *E*. *coli* O157: H7 at all the evaluated concentrations (2xMIC, 1xMIC, and 0.5xMIC). At 24 hour, the inhibitory effect was maintained with 2xMIC and 1xMIC, whereas in wells treated with 0.5xMIC only the Ib-M1 peptide and the antibiotics maintained a percentage of *E*. *coli* growth inhibition higher than 63% (Fig 2).

**Fig 3:**
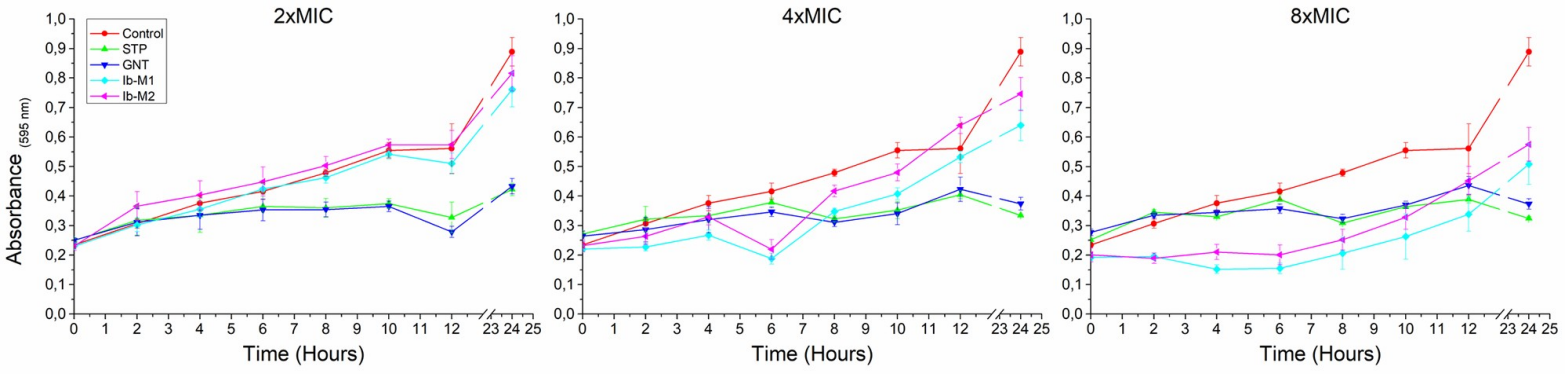
Growth kinetics of *E*. *coli* O157:H7 with Ib-M peptides in log phase. Each concentration was evaluated in quadruplicate. Results are expressed in terms of the arithmetic average ± standard deviation. Data representative of two independent experiments with similar results is shown. **STP:** Streptomycin, **GNT:** Gentamicin.

In log phase, Ib-M1 and Ib-M2 showed an inhibitory effect against *E*. *coli* O157: H7 in the first 8 hours of exposure to 8xMIC; after 24 hours absorbance between 0.5 and 0.6 was obtained in the wells treated with 8xMIC. Wells exposed to 4xMIC only showed growth inhibition during the first 6 hours of peptide exposure. The growth kinetics of *E*. *coli* in the presence of 2xMIC and 1xMIC of Ib-M peptides showed no differences with the growth kinetics of the control. *E*. *coli* during the 24 hours of exposure to the reference antibiotics showed growth inhibition at all the evaluated concentrations (Fig 3). Wells treated with peptides Ib-M4, Ib-M5, and Ib-M6 at 0.5xMIC, 1xMIC and 2xMIC showed similar behavior than the growth control group.

#### Time-kill kinetics

**Fig 4:**
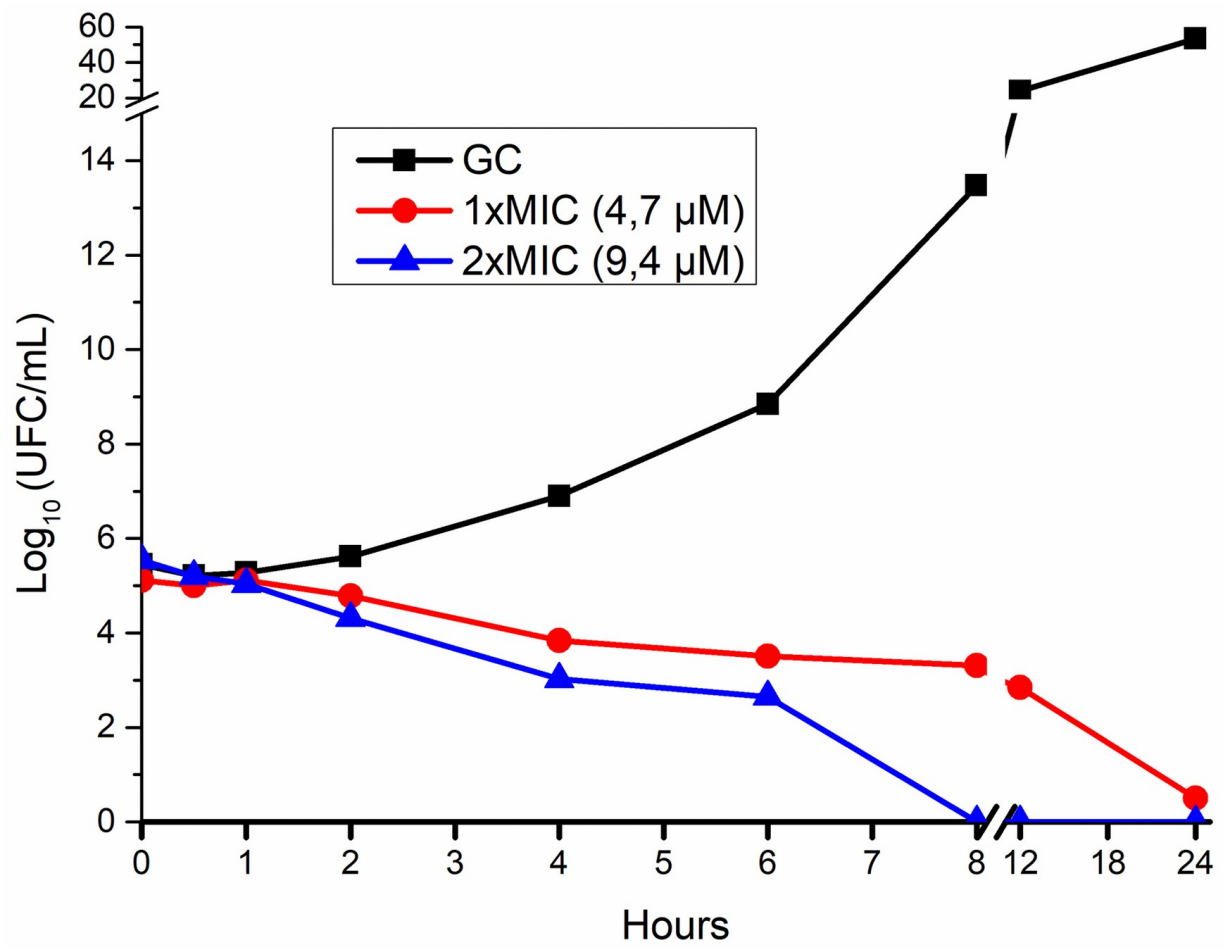
Time-kill kinetics of *E*. *coli* O157: H7 exposed to Ib-M1 peptide. Time-kill kinetics of *E*. *coli* O157: H7 (ATCC 43888) within 24 hours of exposure to Ib-M1. Each concentration was evaluated in quadruplicate. The results are expressed in terms of the arithmetic average ± standard deviation. (The data that do not show error bars correspond to those in which standard deviation is too small to be seen). **GC:** Growth Control.

The bacterial killing was performed only with Ib-M1 since it showed higher activity in the MIC assay of both the reference strain and the clinical isolate. A reduction equivalent to 99.9% (>3 Log10 of CFU/mL) was observed in the number of CFU/mL of *E*. *coli* O157: H7 after 8 hours of exposure to Ib-M1 at 2xMIC, whereas at 1xMIC it was only observed at 24 hours of exposure (Fig 4).

### Cytotoxicity

A cytotoxicity assay was conducted to explore the harmlessness of Ib-M peptides to eukaryotic cells. VERO cells exposed to the Ib-M peptides displayed a CC_50_ in the range from 310.9 to >400 μM, indicating cytotoxicity only at exceedingly large peptides concentrations. Peptides Ib-M4 and Ib-M1 exhibited the lowest toxicity (CC_50_ > 400 ± 0.0 μM and CC_50_ = 395.2 ± 18.3 μM, respectively) while Ib-M2 caused the highest toxicity (CC_50_ = 197, 5 ± 18.3 μM) in VERO cells. The CC_50_ for both reference antibiotics was >3200 μM (Table 3).

**Table 3:** 50% cytotoxic concentrations (CC_50_) and selectivity index (SI) of Ib-M peptides.

Compounds	CC_50_ (μM ± S.D.)	SI	
	VERO Cells	*E*. *coli* O157: H7	
		ATCC 43888	AC188
**Ib-M1**	395.2 ± 18.3	84.1	247
**Ib-M2**	197.5 ± 2.4	42	54.9
**Ib-M4**	>400 ± 0.0	>32	>63.5
**Ib-M5**	315.3 ± 49.1	38	67.1
**Ib-M6**	310.9 ± 4.4	33.1	66.1
**STP**	>3200 ± 0	>340.4	>48
**GNT**	>3200 ± 0	>1777.8	>1391.3

Each concentration was evaluated in triplicate in two independent experiments. The results are expressed in terms of the arithmetic average of each group ± standard deviation (S.D). **STP:** Streptomycin, **GNT:** Gentamicin.

### Selectivity index

Among the evaluated peptides, Ib-M1 displayed the highest SI value of 84.1 and 247 for *E*. *coli* O157: H7 ATCC 43888 and strain AC188 respectively. Ib-M4, Ib-M5 and Ib-M6 exhibited SI values in the range from 32 to 42 in *E*. *coli* O157: H7 ATCC 43888, and 54.9 to 67.1 in *E*. *coli* AC188 (Table 3).

The SI of GNT was 55 times higher than the SI of Ib-M peptides due to low cytotoxicity in VERO cells (>3200 μM). The IS in STP was > 340 with *E*. *coli* O157: H7 ATCC 43888, while with strain AC188 it was reduced to > 48 (Tables 2 and 3).

## Discussion

The activity of Ib-M peptides against *E*. *coli* has been mainly associated with the increase in their positive charge produced by inserting arginine (Arg) residues, as well as with the modification of their hydrophobicity caused by the insertion of tryptophan (Trp) residues [7]. The relationship between the antimicrobial effect and a higher proportion of Arg and Trp residues in AMPs has been previously documented, and it has been reported that these residues can generate cation-pi interactions facilitating the insertion of the peptides into the bacterial cell membrane [18,19]. The secondary α-helical type structures formed by the Ib-M peptides in SDS mimic the conformational changes that the peptides undergo in the presence of the bacterial membrane Likewise, The helicity shown by Ib-M peptides in SDS could be associated with an increase in cation-pi interactions between Arg and Trp residues. Hence, the degree of peptide helicity that has been frequently correlated with a greater antimicrobial activity could favor the bactericidal effect of Ib-M against *E*. *coli* O157: H7 [20,21].

Ib-M1 and Ib-M2 had a higher inhibitory effect against *E*. *coli* O157: H7 than other Ib-M peptides. These results differ from those reports by Flórez et al [7], where Ib-M6 presented the highest activity with inhibitory concentration 50 (IC_50_) of 1 μM against *E*. *coli* K-12.

This difference was to be expected if it takes into account that the genomes of *E*. *coli* K-12 and O157: H7 are considerably different [22], therefore, regulation, gene expression, and metabolic processes in each strain have diverse responses to physiological states of adaptation, growth and survival and to stress conditions [23,24].

The potential of Ib-M peptides was reflected in the inhibition ranges obtained between 1.6 to 12.5 μM, whose values were similar to the *in vitro* activity reported against *E*. *coli* by other AMPs of interest, such as lactoferricin (MIC: 2 μM), and magainin 2 (MIC:8 μM) [19,25]. Ib-M peptides also showed MIC values similar to the antibiotics evaluated with the reference strain ATCC 43888 and presented a higher inhibitory effect than STP with the clinical isolate AC188.

*E*. *coli* O157: H7 AC188 was less susceptible to STP than ATCC 43888; this is correlated with previous reports that have shown the development of resistance of clinical isolates to aminoglycosides because of indiscriminate use of antimicrobials [10,26].

Unlike that observed with STP, the Ib-M peptides showed similar MICs in both strains of *E*. *coli* O157: H7. In this regard, AMP have been considered appropriate molecules to replace antibiotics as they are less prone to develop microbial resistance and their mechanisms of action are different from conventional antibiotics [27].

The bactericidal effect of the peptides was evidenced by the results of the MBC whose values did not exceed those obtained in the MIC by more than two dilutions [13,28]. Likewise, in time-kill kinetics, after *E*. *coli* O157: H7 was exposed for 8 hours to Ib-M1 with a 2xMIC, its bacterial population was reduced by more than 99.9%, and no CFU was observed at 24 hours of exposure. The time required by Ib-M1 to eliminate *E*. *coli* O157: H7 was lengthened, as compared to the kill kinetics other AMP causing the death of 100% of the bacteria in the first 2 hours of exposure due to the increase in the permeability of the *E*. *coli* membrane [14,29]. In the case of Ib-M, the factors that may be associated with the time required by the peptide to eliminated all the viable cells are not known yet. This could be related to one or both of the mechanisms of action used; as it is known, the AMPs can be divided into two groups based on the effect caused in the microorganism: i) Membrane dysfunction due to disruption of the phospholipid bilayer and/or ii) interaction with intracellular targets causing interference in critical metabolic processes, such as DNA, RNA and protein synthesis, as well as in enzymatic activity [30,31].

*E*. *coli* O157: H7 was more susceptible to Ib-M peptides in the latency phase than in the logarithmic phase, since Ib-M needed an 1xMIC in the lag phase and 8xMIC in the log phase to inhibit *E*. *coli*. This result may be due to the different metabolism of the microorganism in each of its growth phases. In the lag phase the microorganism adapts to a new environment, synthesizes new components, there is no cell replication, and prepares for cell division [32], while in the exponential phase it grows at a high rate under optimal conditions of temperature, availability of nutrient and oxygen [33]. Another factor associated with Ib-M activity in the two growth phases would be the quantitative relationship between the concentration of the peptide and the number of bacteria; since the concentration of the inoculum in the lag phase was 5x10^5^ CFU / mL while in the log phase it was 1x10^8^ CFU / mL. Previous reports have shown that the MIC of antimicrobials can increase with a higher cell density, due to factors such as the amount of the compound available for each bacteria, the binding of the antimicrobial to cellular detritus or their denaturation by enzymatic action [34,35].

The low cytotoxicity of Ib-M peptides on VERO cells was reflected in a CC_50_ higher than 197 μM and SI between 32 and 247; these results agree with the low hemolytic activity previously reported [7]. In the same way as the antimicrobial activity, the low toxicity of AMPs can be associated with multiple factors such as charge, sequence, length, and hydrophobicity. One of the most distinctive characteristics of cationic peptides is their high affinity for prokaryotic cell membranes due to their negative charge; while in eukaryotic cells, cationic peptides decrease their affinity with the cytoplasmic membrane for the presence of neutral phospholipids and cholesterol [31,36].

## Conclusion

The *in vitro* antibacterial properties of Ib-M peptides in the pathogenic model of *E*. *coli* O157: H7 have been determined using a reference strain and a clinical isolate. Ib-M1 and Ib-M2 had the highest inhibitory effect, and all the peptides demonstrated bactericidal activity, Ib-M4 being the less effective. All Ib-M peptides exhibited an α-helical type structure in environments that simulate the bacterial membrane. All of them had a low cytotoxic effect on VERO cells. Additional work is necessary to unravel the mechanisms of action involved, as well as studies that allow evaluating the interaction of Ib-M peptides with other antimicrobial compounds.
